# The potential environmental impact of external speakers’ airplane travel to grand rounds conferences

**DOI:** 10.1186/s12940-023-00989-6

**Published:** 2023-04-14

**Authors:** Sally Monahan, Ken Monahan

**Affiliations:** 1 Department of Dermatology, Vanderbilt University Medical Center, Nashville, USA; 2 Division of Cardiovascular Medicine, Vanderbilt University Medical Center, 1215 21st Avenue – Medical Center East 5th floor, Nashville, TN 37232 USA

**Keywords:** Virtual learning, Carbon footprint, COVID-19

## Introduction

The carbon footprint of air-travel to major medical conferences is substantial but as demonstrated during the COVID-19 pandemic, virtual platforms can eliminate these CO_2_ emissions [1, 2]. Grand Rounds conferences are comparatively smaller-scale but higher-frequency events to which external speakers often travel by airplane. Knowledge of the environmental impact of Grand Rounds speakers’ travel and the potential ‘emissions saved’ by using a virtual platform could influence the format of future conferences. For Medicine Grand Rounds (MGR) at our institution, we calculated CO_2_ emissions due to external speaker travel and used this information to project the reduction in emissions if medical centers nationwide conducted Grand Rounds virtually.

## Methods

We studied MGR conferences given before (March 2019-mid-March 2020) and after (mid-March 2020-June 2021) the COVID-19 pandemic, which motivated the transition to a virtual format. We assumed speakers traveled by airplane if the distance between their home institution and ours exceeded 300 miles [1, 2]. To estimate the CO_2_ emissions for each speaker’s air-travel [1, 3], we used the Carbon Footprint Calculator [4] and the International Civil Aviation Organization (ICAO) Carbon Emissions Calculator [5]. Emissions are presented in metric tons (mTon).

## Results

Of the 101 MGR during the observation period, 60 were by external speakers (n = 29 in-person; n = 31 virtual). Due to proximity to our institution, 6 speakers (n = 1 in-person, n = 5 virtual) were excluded. Also excluded were 2 virtual speakers whose home institutions were so distant (Johannesburg South Africa and Paris France) that it is unlikely that they would have traveled to our medical center solely to give MGR. The distance between institutions was 879 ± 553 miles for in-person speakers and 774 ± 373 miles for virtual speakers (p = 0.94; Mann-Whitney test). Using the ICAO calculator, in-person speakers generated a total of 8.6 mTon of CO_2_ emissions and virtual speakers would have generated 9.1 mTon. The Carbon Footprint Calculator estimated total CO_2_ emissions of in-person speakers as 11.7 mTon and that of virtual speakers, had they traveled to give their MGR in-person, as 11.1 mTon.

## Discussion

The CO_2_ emissions associated with external speaker travel for MGR at our institution are approximately 10 mTon per year, a small amount relative to international conferences [1, 2], though still equivalent to emissions from nearly 25,000 miles driven by an average gasoline-powered automobile [6]. However, the prevalence and frequency of MGR and similar conferences, which occur at academic medical centers throughout the country, suggest an even greater impact. Our Department of Medicine includes more than 10 divisions, each with their own Grand Rounds offerings, suggesting that air-travel to those conferences may increase emissions by an additional order of magnitude. The Departments of Surgery and Pediatrics have analogous divisional and Grand Rounds structures, which could plausibly increase Grand Rounds-related emissions 3-fold. There are more than 150 allopathic medical schools in the United States (nearly 200 total medical schools) and most have an affiliated medical center. Thus, the CO_2_ emissions attributable to external Grand Rounds speakers at US academic medical centers may approach 45,000–60,000 mTon per year, similar to the estimated annual travel-related emissions for all residency interviews [3] and comparable to the annual electricity use of more than 10,000 homes [6].

This analysis has several limitations, including those inherent in extrapolating our core result. The observation period is relatively short, which may further limit the accuracy of these estimates. CO_2_ generated by the technology needed to facilitate virtual conferences was not included and may offset some of the benefit. More departments than those included in our analysis likely host external speakers and thus we may have underestimated emissions.

In summary, exclusively virtual presentations by external Grand Rounds speakers could reduce annual CO_2_ emissions substantially.

## Data Availability

Data are available from corresponding author on reasonable request.

## Abbreviations

CO_2_: carbon dioxide
ICAO: international civil aviation organization
MGR: Medicine Grand Rounds
mTon: metric ton
